# Estimated dietary flavonoid intake and major food contributors in the Portuguese population: results from the national food, nutrition and physical activity survey (IAN-AF 2015–2016)

**DOI:** 10.1017/S0007114525000078

**Published:** 2025-02-28

**Authors:** Sofia Martins, Manuela Meireles, Estela Caetano, Daniela Correia, Catarina Carvalho, Catarina Campos Silva, Vânia Magalhães, Sofia Vilela, Carla Lopes, Duarte Torres

**Affiliations:** 1 EPIUnit - Unidade de Investigação Epidemiológica, Instituto de Saúde Pública da Universidade do Porto, Porto, Portugal; 2 Laboratório para a Investigação Integrativa e Translacional em Saúde Populacional (ITR), Porto, Portugal; 3 Departamento de Ciências da Saúde Pública e Forenses e Educação Médica, Faculdade de Medicina da Universidade do Porto, Porto, Portugal; 4 Centro de Investigação da Montanha, Instituto Politécnico de Bragança, Bragança, Portugal; 5 Faculdade de Ciências da Nutrição e Alimentação da Universidade do Porto, Porto, Portugal

**Keywords:** Flavonoids, Intake, Dietary, Food contributors, Database, Dietary survey

## Abstract

Flavonoids are a key class of polyphenols, i.e., phytochemical compounds present in foods and beverages, which have been described as having health benefits in preventing several chronic diseases. Estimating flavonoid intake has already been conducted in several countries but has yet to be performed in Portugal. This study included 5005 participants aged 3–84 years and aimed to estimate dietary flavonoid intake in the Portuguese population, using data from the National Food and Physical Activity Survey 2015–2016, providing information on intake, main food contributors and the socio-demographic factors associated with the intake. Food intake data from the survey was converted to flavonoid intake using a database built to include the most updated USDA databases on flavonoids, isoflavones and proanthocyanidins and the Phenol-Explorer database. The rationale for combining food consumption data and different flavonoid databases using the FoodEx2 classification system was established. Linear regressions assessed the associations between socio-demographic factors and dietary flavonoid intake. The total flavonoid intake of the Portuguese population was estimated to be 107·3 mg/d. Flavanols were the most representative subclass, followed by flavonols, anthocyanidins, flavanones, flavones and isoflavones. Fruits and vegetables were the primary food contributors, providing 31·5 % and 12·4 % of the total flavonoid intake. Adolescents had the lowest total flavonoid intake, and older adults had the highest. This study provides information on the Portuguese population’s dietary flavonoids, allowing for international comparisons. It can also streamline forthcoming investigations into the link between flavonoid consumption and its impact on health, contributing to the future establishment of dietary reference values.

Polyphenols are bioactive compounds present in plant-based foods and beverages synthesised by plants’ secondary metabolism, encompassing a wide range of subclasses based on their distinctive chemical structures. These subclasses include phenolic acids, flavonoids, tannins, coumarins, lignans, quinones, stilbenes and curcuminoids. Each of these classes exhibits unique properties and bioactive effects, contributing to the diverse health benefits associated with polyphenols. Among them, flavonoids are the largest and most studied class^(1)^. Epidemiological studies have shown that a higher intake of flavonoids is associated with a reduced risk of several chronic diseases, including cardiovascular diseases^(2)^, type 2 diabetes^(3)^, cognitive dysfunction^(4)^ and several types of cancer^(5)^. These potential health benefits are attributed to their strong antioxidant capacity, anti-inflammatory effects, anticarcinogenic and antimicrobial properties^(6–10)^.

Flavonoids can be classified into different subclasses according to their chemical structure. Six of these subclasses have received special attention from the scientific community: (i) flavonols (e.g., kaempferol, quercetin), which can be found in onions, leeks and broccoli; (ii) flavones (e.g., apigenin, luteolin), found in parsley and celery; (iii) isoflavones (e.g., daidzein, genistein), mainly found in soy and soy products; (iv) flavanones (e.g., hesperetin, naringenin), mainly found in citrus fruit and tomatoes; (v) flavanols (e.g., (+)-catechin, (−)-epicatechin, epigallocatechin, epigallocatechin gallate (EGCG)), abundant in green tea, red wine and chocolate and (vi) anthocyanidins (e.g., pelargonidin, cyanidin, malvidin), whose sources include red wine and berry fruits^(11–13)^.

The concept of establishing dietary reference intakes (DRI) for flavonoids was raised a few years ago. The scientific community argues that, like nutrients, flavonoids should be considered essential due to their functional role in promoting lifespan. Consequently, there is a proposition to classify flavonoids as ‘lifespan essential’ due to their significant contributions to overall health and well-being^(14)^.

Population-based estimation of dietary flavonoid intake, based on reliable food databases, is the first step to determining the dietary requirements, followed by the ability to quantify the consumption of these bioactive compounds needed to exert health benefits^(15,16)^. The estimation of flavonoid intake has been performed in several countries worldwide, as reviewed by Del Bo and colleagues^(15)^, but it has yet to be undertaken in Portugal. Del Bo’s review highlights substantial regional variations in polyphenol intake, with Japan reporting the highest intake (∼1500 mg/d) and countries such as Spain showing much lower levels (∼300 mg/d). Flavonoid intakes also vary widely, with Poland and Australia reporting the highest (∼600 mg/d) and China and Korea reporting the lowest (∼60 mg/d). Various factors may shape these differences, including dietary habits, food sources, cultural preferences, geographical regions, socio-demographic characteristics, dietary assessment methods and the choice of food composition databases^(15)^.

This study aims to accurately estimate the dietary flavonoid intake within the Portuguese population using consumption data from the National Food, Nutrition, and Physical Activity Survey 2015–2016 (IAN-AF 2015–2016). It combines this data with a compiled flavonoid database coded according to FoodEx2 standards, integrating various publicly available flavonoid databases. Furthermore, this study also aimed to explore socio-demographic and lifestyle factors influencing flavonoid intake and its main food contributors.

## Methods

### Participants

The present study used data from the IAN-AF 2015–2016. A comprehensive description of the study design has been previously published^(17)^. The IAN-AF 2015–2016 aimed to representatively sample individuals from the general Portuguese population, ranging in age from 3 months to 84 years old. The sampling process involved a multistage approach, targeting each geographical region (NUTSII). The IAN-AF 2015–2016 research protocol received approval from the National Commission for Data Protection, the Ethical Committee of the Institute of Public Health of the University of Porto and the Ethical Commission of each Regional Administration of Health. Written informed consent was obtained from all participants, and in the case of minors, their parents or legal caregivers provided the informed consent on their behalf. A total of 5811 participants completed two computer-assisted face-to-face interviews conducted by trained nutritionists either at primary healthcare units or in participants’ homes. The response rate among eligible participants was 35 %, with higher participation rates observed among children and adolescents (approximately 46 %) and lower rates among older adults (approximately 20 %). Due to the substantial differences in dietary patterns in children under 3 years old, these participants were excluded from this analysis. As a result, the final sample comprised 5005 participants.

### Dietary assessment

The IAN-AF 2015–2016 survey collected data over 12 months, from October 2015 to September 2016, to account for seasonal variability of food consumption^(17)^. The data were collected by trained interviewers using an electronic platform – You eAT&Move and dietary intake was estimated based on the eAT24 (Electronic Assessment Tool for 24-hour recall) software^(18)^. This software integrates the Portuguese Food Composition Table and international data to fill in missing values. It allows the collection of detailed information on the quantification of foods, recipes and supplements reported from 24-hour dietary recalls and food diaries.

Dietary intake data from children under 10 years old were collected using two non-consecutive 1-day food diaries filled out by their primary caregiver(s). These diaries were completed on separate days, with an interval of 8–15 days between them. A face-to-face interview with parents or other caregivers was conducted to gather supplementary information regarding food descriptions and quantities. For participants in other age groups, two non-consecutive 24-hour recalls were used to obtain dietary intake data, where a caregiver was present during the administration process. 24-hour recalls were also conducted with an 8–15-day interval between them.

Food portions were calculated using the determined weight or volume, household measurements or estimated using a picture book^(19)^ based on a previously validated one^(20)^. All reported foods and beverages were then categorised into groups and subgroups, facilitating the subsequent analysis process and classified accordingly with the detailed European Food Safety Authority (EFSA) FoodEx2 classification system^(21)^. Finally, the intake of different foods and beverages was estimated using the mean of the 2-day dietary assessments.

### Other variables

As part of the survey, participants provided information on various aspects, including socio-demographic characteristics and lifestyle variables. Therefore, this study focused on the following socio-demographic variables: sex (‘female’ or ‘male’), age group (‘children’, ‘adolescents’, ‘adults’ or ‘older adults’), education level (‘none, 1^st^ and 2^nd^ cycle’, ‘3^rd^ cycle and high school’ or ‘higher education’) and degree of urbanisation of the area of residence (predominantly urban, mostly urban, or predominantly rural). The highest level of parental education completed was considered for children and adolescents. The lifestyle variable included was regular physical activity (‘yes’ or ‘no’). Regular physical activity excluded school activities for children, while adults self-reported regular physical activity practices.

### Flavonoid database

To date, a flavonoid database specific to the Portuguese diet is not available; therefore, for the present study, a flavonoid database was constructed based on the USDA Database for the Flavonoid Content of Selected Foods (release 3·3)^(22)^, the USDA Database for the Isoflavone Content of Selected Foods (release 2·1)^(23)^, the USDA Database for the Proanthocyanidin Content of Selected Foods (release 2)^(24)^ and the Phenol-Explorer (PE) database (version 3·6)^(25)^. These databases represented the most comprehensive flavonoid and polyphenols databases freely available.

Flavonoids in USDA databases are presented as aglycone equivalents, while in the PE, glycosylated molecules (as found in nature) are presented^(26)^. A correction factor was applied to glycosylated molecules in PE. In cases where the flavonoid was conjugated with a phenolic acid, it was also considered just the aglycone form of the flavonoid, excluding the phenolic acid. Particularly, the flavanols (or flavan-3-ols) conjugated with gallic acid, presented in USDA databases: (–)-Epicatechin 3-gallate, (–)-Epigallocatechin 3-gallate and (+)-Gallocatechin 3-gallate were corrected to their aglycone forms: Epicatechin, (–)-Epigallocatechin and (+)-Gallocatechin, respectively. This correction factor was calculated considering the ratio between the molecular weight of the aglycone form and the glycosylated or conjugated form. The respective aglycone form of each compound was confirmed on the PE website, and molecular weights of glycoside and aglycone forms were retrieved from either PE or PubChem^(27)^. In the few cases with a difference, extended research was done to find the best reasonable match. For instance, the molecular weight of cyanidin-3-glucoside is 449·38 g/mol, while its aglycone, cyanidin, is 287·24 g/mol. By calculating the ratio 287·24/449·38, we derive the correction factor of 0·639. This correction factor was applied (multiplied) to the total amount of cyanidin-3-glucoside obtained. This calculation was conducted individually for each molecule^(22)^. Additionally, the arithmetic mean of the values from the two databases was used for food items that appeared in both databases with different flavonoid values.

A unique code was attributed to each food item presented in the merged flavonoid database (USDA + PE) according to the FoodEx2 system. The FoodEx2 classification system, developed by EFSA, standardises food classification by organising individual food items into groups and broader categories, with a hierarchical parent–child relationship^(21)^. When a food item from the food database containing all the foods reported in the IAN-AF 2015–2016 did not have a corresponding match at the merged flavonoid database, the arithmetic mean of the closest parents’ values was applied. Some logical zeros were attributed to some food groups to avoid cases where closest parents did not have data on flavonoid composition and prevent the attribution of values distant in the hierarchical FoodEx2 tree. These zero values were assigned to the animal food groups meat, fish, eggs and dairy, except for dairy containing fruits or chocolate. Zero values were also assigned to flavonoid compounds in the subclasses that were not expected to be present in a particular food or food group, as previously done by USDA databases^(28)^. Since chocolate is a flavonoid-rich ingredient, some food items containing chocolate were deconstructed. It was considered 15 % for ice creams, 7 % for cereals and 3 % for cakes containing chocolate. These values were chosen, considering the labelling of common Portuguese brands. A total of 888 FoodEx2 base terms, corresponding to the same number of food items, were compiled into the IAN-AF 2015–2016 flavonoid dietary data. Of these, 258 food items were directly retrieved from the merged flavonoid database (USDA + PE), with 124 items found in USDA and PE, 103 only in USDA and 31 only in PE. The remaining 630 items were assigned values using the FoodEx2 hierarchical tree system.

### Statistical analysis

Total flavonoids and their main subclasses were estimated in milligrams per day. Geometric mean intakes of total flavonoids (and their subclasses) with respective 95 %CI were calculated by sex, age group, education level, physical activity and area of residence. T-tests and Wald tests evaluated overall dietary flavonoid intake differences across the socio-demographic and lifestyle variables. These intakes were expressed as milligrams of dietary flavonoids per 1000 kcal to adjust for variations in total energy intake. Adjusted linear regression models were fitted for log-transformed total flavonoids and their main subclasses. Results were interpreted as relative mean differences and respective 95 %CI. Models were stratified by sex and adjusted for age group, education level, physical activity, area of residence and total energy intake. The percentage contribution of each flavonoid subclass intake to the total intake of flavonoids was calculated. The statistical analysis conducted in this study was weighted according to the complex sampling design used in the IAN-AF 2015–2016, as described in detail elsewhere^(17)^. The R software version 3·4·0 for Windows was used, and a significance level of 5 % was assumed.

## Results

### Sample characterisation

The sample was characterised by sex, age group, education level, physical activity and area of residence, as shown in Table 1. The percentages of female and male participants were very close (51·2 % *v*. 48·8 %). Stratifying by age, 597 (6·9 %) of the participants were between 3 and 9 years old (children), 616 (8·4 %) between 10 and 17 years old (adolescents), 3091 (68·1 %) between 18 and 64 years (adults) and 701 (16·6 %) between 65 and 84 years old (older adults). Almost half of the population (45·8 %) reported having completed the third cycle and high school, followed by the ones reporting none, first and second cycle (29·7 %) and higher education (24·5 %). Most of the sample reported not having regular physical activity (57·6 %) and lived in a predominantly urban area (77·6 %).

**Table 1.** Energy-Adjusted mean daily dietary intake of total flavonoids and flavonoid subclasses (mg/d) by participants’ characteristics, weighted for the complex survey design

	*n*	%	Total flavonoids			Flavanols			Flavonols			Flavones			Anthocyanidins			Flavanones			Isoflavones		
			EA-GM	95 % CI	*P*-value	EA-GM	95 % CI	*P*-value	EA-GM	95 % CI	*P*-value	EA-GM	95 % CI	*P*-value	EA-GM	95 % CI	*P*-value	EA-GM	95 % CI	*P*-value	EA-GM	95 % CI	*P*-value
Total	5005	100·0	107·3	103·3, 111·3		49·6	46·3, 53·2		13·6	13·1, 14·1		2·8	2·7, 3·0		2·8	2·4, 3·2		0·3	0·2, 0·4		0·2	0·1, 0·2	
Sex																							
Female	2613	51·2	104·2	98·6, 110·1	0·092	49·0	44·1, 54·4	0·641	14·7	14·1, 15·4	**<0·001**	3·4	3·2, 3·6	**<0·001**	1·8	1·4, 2·2	**<0·001**	0·2	0·1, 0·3	**<0·001**	0·2	0·2, 0·2	**<0·001**
Male	2392	48·8	110·6	105·6, 115·8		50·3	46·9, 53·9		12·4	11·8, 13·1		2·3	2·1, 2·5		4·4	3·5, 5·3		0·5	0·3, 0·6		0·1	0·1, 0·1	
Age group																							
Children	597	6·9	94·6	87·9, 101·8	**<0·001**	61·6	55·9, 67·9	**<0·001**	10·5	9·8, 11·3	**<0·001**	1·7	1·5, 1·9	**<0·001**	1·1	0·7, 1·7	**<0·001**	0·1	0·0, 0·2	**<0·001**	0·1	0·1, 0·1	**<0·001**
Adolescences	616	8·4	79·3	72·6, 86·6		45·2	39·6, 51·6		8·7	8·1, 9·4		1·9	1·7, 2·1		0·4	0·3, 0·6		0·1	0·0, 0·1		0·1	0·1, 0·1	
Adults	3091	68·1	106·0	101·3, 110·9		47·1	43·7, 50·8		13·9	13·3, 14·6		3·0	2·8, 3·2		3·1	2·6, 3·7		0·4	0·3, 0·5		0·2	0·2, 0·2	
Older adults	701	16·6	138·2	123·9, 154·2		58·9	46·7, 74·2		16·7	15·5, 18·0		3·4	3·0, 3·8		6·9	4·3, 10·9		0·5	0·3, 1·0		0·1	0·1, 0·2	
Education level																							
None, first and second cycle	1497	29·7	123·0	115·3, 131·2	**<0·001**	52·5	47·9, 57·5	0·383	15·9	15·2, 16·7	**<0·001**	2·9	2·7, 3·2	**0·009**	5·1	3·9, 6·8	**<0·001**	0·4	0·3, 0·6	0·357	0·1	0·1, 0·1	**<0·001**
Third cycle and high school	2201	45·8	98·5	92·8, 104·6		47·6	43·3, 52·2		12·3	11·6, 13·0		2·6	2·4, 2·8		1·8	1·5, 2·3		0·3	0·2, 0·4		0·2	0·1, 0·2	
Higher education	1291	24·5	106·6	96·8, 117·4		49·9	39·6, 62·9		13·4	12·6, 14·2		3·1	2·8, 3·4		2·8	2·0, 4·0		0·3	0·1, 0·5		0·2	0·2, 0·2	
Regular physical activity (yes/no)																							
Yes	2055	42·4	110·8	105·9, 115.1	0·138	55·7	52·6, 59·1	**0·003**	13·6	12·9, 14·5	0·707	2·9	2·7, 3·2	0·226	2·6	2·0, 3·2	0·417	0·3	0·2, 0·5	0·340	0·2	0·2, 0·2	**0·001**
No	2878	57·6	104·9	99·2, 110·9		45·7	40·7, 51·2		13·5	12·9, 14·1		2·7	2·5, 2·9		3·0	2·4, 3·7		0·3	0·2, 0·4		0·1	0·1, 0·2	
Geographical region type																							
PUA	3650	77·6	106·6	102·0, 111·5	0·832	50·0	46·0, 54·3	0·683	13·3	12·7, 13·9	0·051	2·9	2·8, 3·1	0·066	2·7	2·3, 3·2	0·191	0·3	0·2, 0·4	0·674	0·2	0·2, 0·2	0·125
MUA	863	13·8	108·8	100·7, 117·7		50·8	47·5, 54·3		14·9	13·7, 16·2		2·5	2·1, 2·9		2·4	1·5, 3·6		0·4	0·2, 0·6		0·1	0·1, 0·2	
PRA	492	8·5	110·7	95·8, 127·8		44·8	33·9, 59·2		14·1	12·7, 13·9		2·5	2·2, 2·9		4·0	2·6, 6·2		0·4	0·2, 0·9		0·1	0·1, 0·2	

Abbreviations: EA-GM, energy-adjusted geometric mean; PUA, predominantly urban area; MUA, moderately urban area; PRA, predominantly rural area. Significant values are in bold.

### Total and subclasses of dietary flavonoid intake

In this representative sample of the Portuguese population, the estimated energy-adjusted geometric mean intake of total dietary flavonoids was 107·3 mg/d (Table 1). Females had higher intakes of flavonols, flavones and isoflavones, whereas males had higher intakes of anthocyanidins and flavanones. The highest intake of total flavonoids was found among older adults (138·2 mg/d) and the lowest in adolescents (79·3 mg/d). The intake of most classes of flavonoids was influenced by age group. For flavanols, children presented the highest and adolescents the lowest intake. Flavonols, flavones, anthocyanidins and flavanones were particularly high among adults and older adults. Regarding anthocyanidins, the older adults presented a particularly high intake compared to the other age groups. Participants with the lowest levels of education (none, first and second cycle) had the highest intakes of total flavonoids (123·0 mg/d), flavonols, anthocyanidins and flavanones. Participants with a higher education level had the highest intakes of flavones and isoflavones. Regular physically active participants had higher intakes of flavanols and isoflavones. No statistically significant differences were found between geographical region types. Energy-unadjusted geometric mean intakes of total dietary flavonoids and main subclasses are presented in online Supplementary Table S1.

### Factors associated with dietary flavonoid intake

Relative mean differences of total dietary flavonoids and main subclass intake, stratified by sex, are presented in Table 2 (for females) and Table 3 (for males).

**Table 2.** Associations between socio-demographic characteristics and the mean daily dietary intake of total flavonoids and subclasses (mg/d) among females, weighted for the complex survey design

	Total Flavonoids		Flavanols		Flavonols		Flavones		Anthocyanidins		Flavanones		Isoflavones	
	RD	95 %CI	RD	95 %CI	RD	95 %CI	RD	95 %CI	RD	95 %CI	RD	95 %CI	RD	95 %CI
Age														
Children	**0·87**	**0·77, 0·98**	**1·21**	**1·02, 1·42**	**0·71**	**0·63, 0·81**	**0·48**	**0·40, 0·57**	0·57	0·28, 1·15	**0·29**	**0·10, 0·83**	**0·56**	**0·37, 0·85**
Adolescences	**0·72**	**0·63, 0·83**	0·90	0·75, 1·09	**0·60**	**0·52, 0·68**	**0·50**	**0·42, 0·6**	**0·15**	**0·07, 0·29**	**0·19**	**0·06, 0·60**	**0·64**	**0·47, 0·88**
Adults	ref		ref		ref		ref		ref		ref		ref	
Older adults	1·12	0·88, 1·44	1·12	0·63, 1·97	1·03	0·88, 1·21	1·18	0·95, 1·47	1·03	0·43, 2·44	1·09	0·34, 3·48	0·98	0·68, 1·40
Education level														
None, 1^st^ and 2^nd^ cycle	0·90	0·70, 1·15	0·96	0·54, 1·71	1·03	0·87, 1·22	0·86	0·72, 1·04	1·08	0·48, 2·40	0·86	0·34, 2·19	**0·71**	**0·51, 0·99**
3^rd^ cycle and high school	0·92	0·78, 1·08	1·03	0·71, 1·49	0·92	0·81, 1·04	0·86	0·73, 1·01	0·62	0·32, 1·20	0·88	0·36, 2·14	0·81	0·62, 1·05
Higher education	ref		ref		ref		ref		ref		ref		ref	
Regular physical activity (yes/no)														
Yes	ref		ref		ref		ref		ref		ref		ref	
No	**0·79**	**0·71, 0·89**	**0·69**	**0·55, 0·87**	**0·82**	**0·74, 0·90**	**0·80**	**0·71, 0·91**	0·77	0·48, 1·23	0·65	0·30, 1·39	**0·74**	**0·60, 0·91**
Geographical region type														
PUA	ref		ref		ref		ref		ref		ref		ref	
MUA	0·98	0·89, 1·09	0·99	0·87, 1·13	1·08	0·99, 1·17	0·86	0·70, 1·06	0·61	0·31, 1·2	1·28	0·65, 2·52	0·90	0·62, 1·29
PRA	0·86	0·66, 1·13	0·72	0·42, 1·23	0·95	0·78, 1·16	**0·70**	**0·56, 0·89**	0·88	0·42, 1·86	0·95	0·35, 2·55	0·85	0·57, 1·27

Abbreviations: RD, relative mean differences; ref, reference category; PUA, predominantly urban area; MUA, moderately urban area; PRA, predominantly rural area. Values are presented as adjusted models (relative differences adjusted for age groups, education level, regular physical activity, geographic region type and energy intake) from the reference group (ref). Significant values are in bold.

**Table 3.** Associations between socio-demographic characteristics and the mean daily dietary intake of total flavonoids and subclasses (mg/d) among males, weighted for the complex survey design

	Total Flavonoids		Flavanols		Flavonols		Flavones		Anthocyanidins		Flavanones		Isoflavones	
	RD	95 %CI	RD	95 %CI	RD	95 %CI	RD	95 %CI	RD	95 %CI	RD	95 %CI	RD	95 %CI
Age														
Children	**0·86**	**0·76, 0·98**	**1·35**	**1·11, 1·65**	**0·73**	**0·65, 0·83**	**0·63**	**0·52, 0·76**	**0·26**	**0·13, 0·50**	**0·20**	**0·07, 0·53**	**0·64**	**0·45, 0·91**
Adolescences	**0·80**	**0·70, 0·91**	1·01	0·82, 1·25	**0·71**	**0·63, 0·81**	**0·83**	**0·71, 0·96**	**0·10**	**0·06, 0·19**	**0·13**	**0·06, 0·31**	**0·55**	**0·40, 0·75**
Adults	ref		ref		ref		ref		ref		ref		ref	
Older adults	**1·39**	**1·27, 1·52**	**1·37**	**1·18, 1·59**	**1·12**	**1·00, 1·25**	1·09	0·92, 1·29	**4·35**	**2·90, 6·53**	**3·85**	**1·86, 7·98**	1·24	0·87, 1·76
Education level														
None, first and second cycle	**1·19**	**1·03, 1·39**	1·19	0·93, 1·52	1·07	0·95, 1·21	**0·76**	**0·63, 0·91**	1·62	0·88, 2·96	2·15	0·78, 5·88	**0·50**	**0·36, 0·68**
Third cycle and high school	0·94	0·82, 1·07	0·98	0·77, 1·24	0·91	0·83, 1·01	**0·80**	**0·68, 0·94**	0·62	0·36, 1·07	1·72	0·70, 4·20	0·96	0·70, 1·32
Higher education	ref		ref		ref		ref		ref		ref		ref	
Regular physical activity (yes/no)														
Yes	ref		ref		ref		ref		ref		ref		ref	
No	1·01	0·91, 1·12	0·92	0·78, 1·08	1·01	0·92, 1·11	0·97	0·84, 1·12	1·20	0·77, 1·88	0·74	0·41, 1·32	**0·79**	**0·62, 1·00**
Geographical region type														
PUA	ref		ref		ref				ref		ref			
MUA	1·05	0·93, 1·18	1·04	0·91, 1·19	**1·21**	**1·08, 1·36**	0·93	0·78, 1·11	1·04	0·62, 1·73	1·13	0·60, 2·13	0·82	0·60, 1·12
PRA	**1·20**	**1·08, 1·33**	**1·14**	**1·00, 1·29**	**1·19**	**1·06, 1·35**	1·16	0·99, 1·36	**1·90**	**1·00, 3·59**	1·60	0·53, 4·88	0·99	0·71, 1·37

Abbreviations: RD, relative mean differences; ref, reference category; PUA, predominantly urban area; MUA, moderately urban area; PRA, predominantly rural area. Values are presented as adjusted models (relative differences adjusted for age groups, education level, regular physical activity, geographic region type and energy intake) from the reference group (ref). Significant values are in bold.

Children and adolescents had lower intakes of total dietary flavonoids than adults among females and males. Adolescents exhibited the lowest flavonoid consumption, with females and males consuming 28 % and 20 % less total flavonoids, respectively, compared to adults. Female children had 13 % lower intake than female adults. Male children had 14 % lower intakes, and male older adults had a 39 % higher intake than male adults.

In both sexes, children and adolescents had lower intakes of the six classes than adults, apart from anthocyanidins in female children and flavanols in adolescents, where the relative mean differences were not statistically significant. The older adult males had higher intakes of flavanols (37 %), flavonols (12 %), anthocyanidins (335 %) and flavanones (285 %) than adult males. Compared with the higher educated, males with lower levels of education had a 19 % higher intake of total flavonoids and females with lower education had a 29 % lower intake of isoflavones. Compared to regular physically active, the non-regular physically active participants had lower intakes of total flavonoids, flavanols, flavonols, flavones and isoflavones among females and lower intakes of isoflavones among males. Regarding area of residence, female participants living in predominantly rural areas had 30 % lower intakes of flavones than those living in predominantly urban areas. Male participants in predominantly rural areas had 20 % higher intakes of total flavonoids, 14 % of flavanols, 19 % of flavonols and 90 % of anthocyanidins than those in predominantly urban areas. Males living in moderately urban areas had 21 % higher intakes of flavonols than males living in predominantly urban areas.

### Dietary flavonoid intake and its main food contributors

Dietary flavanols intake was the most representative subclass (54·1 %), followed by flavonols (16·4 %), anthocyanidins (14·6 %), flavanones (8·6 %), flavones (5·1 %) and isoflavones (1·1 %) (Table 4).

**Table 4.** Contributions (%) of each flavonoid subclass to the mean daily intake of total flavonoids

	Percentage (%)	sd
Flavanols	54·1	0·52
Flavonols	16·4	0·34
Anthocyanidins	14·6	0·44
Flavanones	8·6	0·33
Flavones	5·1	0·18
Isoflavones	1·1	0·07

sd, standard deviation.

**Fig. 1.**
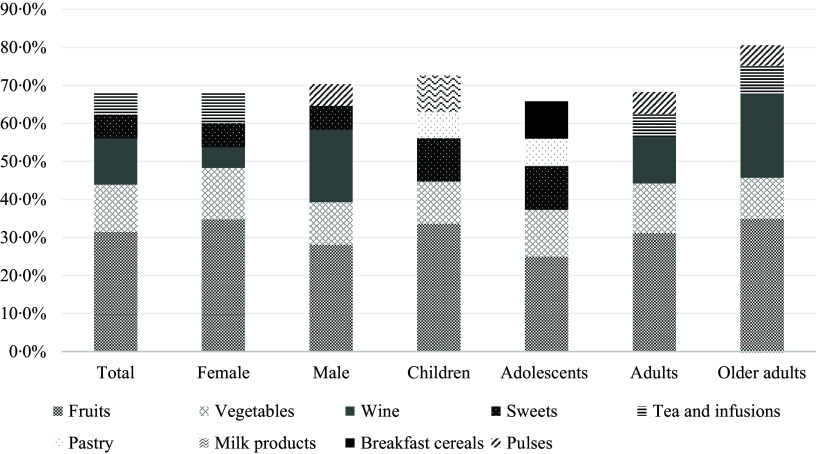
Contributors (%) of each food group to the mean daily intake of total flavonoids.

The fruit and vegetable groups appear as the primary food contributors to the total dietary flavonoid intake, with 31·5 % and 12·4 %, respectively. Wine, sweets (including chocolates and sweets containing chocolate) and tea were among the top five main contributors. The top five food contributors differ according to the age groups of the population. Fruits and vegetables represent a major contribution in females compared to males, while wine contributes more to total flavonoid intake in males (19·1 %) than in females (5·5 %). Sweets make a major contribution to children and adolescents due to chocolate products, while pulses are only among the top five flavonoid contributors in adults and older adults (Fig. 1).

## Discussion

To the best of our knowledge, this study was the first to estimate the dietary flavonoid intake in the Portuguese population. Crossing data from the IAN-AF 2015–2016 and flavonoid content food databases facilitated the assessment of flavonoid consumption in Portugal and its primary food contributors.

The choice of the food databases when assessing flavonoid intake is of major relevance. Discrepancies regarding food availability and flavonoid content between the USDA and the PE databases were found. Therefore, complementarily using the two databases has been suggested to convert food consumption into flavonoid intake more accurately^(15)^. Integrating and harmonising data from different sources allowed for a broader, more robust and reliable dietary assessment of the Portuguese population’s total flavonoids and flavonoid subclasses intake.

This study established a comprehensive approach for integrating various datasets with the FoodEx2 classification system to estimate the dietary flavonoid intake in the Portuguese population. The FoodEx2 classification system served as a valuable tool in this study. It provided a standardised classification for categorising and quantifying flavonoid content in foods and beverages, ensuring consistency and comparability across databases. Therefore, this harmonisation process facilitated the integration and combination of different datasets. Furthermore, integrating this classification system into the dietary databases enabled the estimation of dietary flavonoid intakes for region-specific foods that lacked corresponding entries in the USDA + PE database. This approach was essential to avoid underestimating dietary flavonoid intake in the Portuguese population by incorporating the arithmetic means of flavonoids of the hierarchically closest food items.

Several studies have been conducted in Europe to assess the intake of flavonoids and subclasses nationally. The estimated flavonoid intake varies widely across different populations due to differences in methods of flavonoid estimations, tools used to assess dietary intake, dietary patterns and type of study design. The methodological deviations between studies make an essential limitation in comparing specific values of flavonoid intake. Specifically, our study employed geometric means due to the right-skewed distributions of total flavonoids and their main subclasses. While less common in many studies, this methodological choice provides a more representative measure of the central tendency for skewed data and likely accounts for the considerably lower mean intake values observed in our results compared to studies that reported arithmetic means. Notably, when compared to a recent analysis using the 2017–2018 National Health and Nutrition Examination Survey (NHANES) data, which also utilised geometric means for total flavonoid and subclass intakes, our geometric mean intakes were over twice as high (107·3 mg/d *v*. 45·7 mg/d). Additionally, while our flavonoid subclass intake values were generally closer to those reported in the NHANES study than other studies using arithmetic means, our findings still demonstrated higher intakes across most subclasses, particularly for flavanols, flavonols and flavones^(29)^. In this study, the flavanols subclass was the major contributor to the total dietary flavonoid intake (54·1 %), resulting from fruit consumption as the primary food contributor to the total flavonoid intake. The second major contributor was flavonols, followed by anthocyanidins, flavanones, flavones and isoflavones. In several European and non-European studies, the flavanols subclass has also been reported as the main contributor to the total flavonoid intake, and isoflavones and flavones are the subclasses with the lower contributions^(30–32)^.

Regarding the food groups contributors to the total flavonoid intake, our findings align with previous research, which estimated the intake of flavonoids in several countries and identified the fruits group as the primary contributor to the total flavonoid intake in Mediterranean and non-Mediterranean countries^(33)^. Additionally, a study that estimated the total flavonoid intake in a Mediterranean population highlighted fruits and vegetables as the primary food contributors to flavonoid intake^(34)^. In contrast, a study conducted in the USA identified tea, citrus fruit juices and wine as the primary food sources of flavonoid intake in adults^(30)^. This discrepancy may reflect the distinct dietary patterns and cultural habits between Portugal and the USA. The higher prominence of flavonoids from tea in the USA diet may be attributed to its widespread consumption, whereas tea is less commonly consumed in Portugal. Additionally, citrus fruits are common breakfast items in the USA and contribute significantly to flavonoid intake but are less popular in Portugal, where whole fruits are typically preferred. Despite those differences, both studies have found wine as the third leading source of flavonoid intake, reflecting its role in the Mediterranean (Portuguese) and non-Mediterranean (USA) diets^(35,36)^.

The present study also accounted for variations in flavonoid intake across physical activity and socio-demographic characteristics. A variability of flavonoid intake, specifically differences according to physical activity and age groups, can be seen in our study. The European Prospective Investigation into Cancer and Nutrition (EPIC) study estimated flavanol intake in ten European countries using a 24-hour recall and found that physically active people have higher intakes than physically inactive^(37)^, such as the case of our results for females. A study with participants of the Moli-sani cohort also found higher intakes of each of the six classes of flavonoids among physically active and moderately active participants^(34)^, even though these relate to both sexes. In our study, the higher total dietary flavonoid and most subclasses intake observed among females may reflect healthier lifestyles, characterised by healthier dietary patterns and greater intake of flavonoid-rich foods. Regarding age groups, results from another EPIC study found higher intakes of total flavonoids in older adults than adults^(38)^, and a study conducted on the Australian population also found lower intakes of flavonoid subclasses among children and adolescents^(39)^. These age-related differences regarding dietary flavonoid intake may be related to the adopted dietary patterns, where age plays an important role. Some studies conducted in different populations, including the Portuguese population, have shown a higher adherence to the Mediterranean diet (characterised by flavonoid-rich foods, mainly fruits and vegetables) among older adults^(36,40,41)^. This factor likely explains the higher intake of total flavonoids and most subclasses in older adults. Notably, among males, older adults had 39 % higher intakes of total flavonoids when compared with the reference group (younger adults), and higher intakes of anthocyanidins and flavanones. Regarding the level of education and total flavonoid intake, it was expected that higher-educated participants would follow healthier diets and, therefore, have higher intakes of total flavonoids. However, in this study, the less educated male had a 19 % higher intake of total flavonoids than the higher-educated ones. These findings may be partly explained by the IAN-AF 2015–2016 reported results^(36)^, which identified wine as the most consumed alcoholic beverage in the Portuguese population, with a particularly high intake among males (148 g/d) compared to females (26 g/d). Similarly, another study conducted in the Portuguese population^(42)^ reported the highest wine consumption among less educated and older males^(42)^.

The distribution of food group contributors to total flavonoid intake also varies according to sex and age groups. Even though the fruits group remains the primary contributor across all groups. The highest differences were found in wine, contributing to 19·1 % of the total flavonoid intake among males and 22·1 % in older adults. In comparison, it contributed to only 5·5 % of the intake among females and 12·3 % in younger adults. Another important difference is the contribution of sweets, which are in the top five for children and adolescents, with a contribution of 11·3 % among children and 11·5 % among adolescents. These differences in the contributors reflect the variations in the Portuguese intake of different food groups according to sex and age, as also previously published in the IAN-AF 2015–2016 reported results^(36)^. It is important to highlight that younger participants (children and adolescents) had lower intakes of dietary flavonoids compared to the reference group (female and male adults), and at the same time were the groups having significant contributions of dietary flavonoids coming from ultra-processed foods, particularly sweets and pastry groups in both groups, children and adolescents, and milk products and breakfast cereals in the top five contributors in children and adolescents, respectively. These findings align with a recent cross-sectional study conducted in a representative sample of the USA population, which suggests that the intake of ultra-processed foods is associated with lower intakes of total dietary flavonoids^(29)^.

In this study, several Portuguese foods did not directly match the created database derived from the USDA and PE databases harmonisation; therefore, a similar food was selected to catch the potential flavonoid intake. Cooking methods were not considered due to the limited information available in the databases. In this study, food diaries and 24-hour recalls offer advantages over a Food Frequency Questionnaire (FFQ), which requires a higher record capacity from the participants and may lack a detailed description of food recipes (including food ingredients). However, these methods are still prone to bias and may not reflect the usual intake of bioactive compounds, such as flavonoids. Dietary intake biomarkers offer a more objective and accurate way to measure flavonoid intake by directly measuring flavonoid or their metabolite concentrations in biological samples (e.g., blood or urine). This helps overcome limitations associated with self-report intakes, such as misreporting, recall bias and social desirability bias. However, it may be costly, and availability is limited to specific bioactive molecules^(43,44)^. Additionally, individual variations, such as gut microbiota, can affect the accuracy of biomarker measurements^(7)^. Therefore, while promising, their use remains limited, and intrinsic challenges related to self-reporting may remain unsolved. Combining methods such as FFQ with food diaries or recalls is suggested to enhance accuracy. However, these tools should ideally be validated with flavonoid biomarkers for more reliable dietary intake estimates^(45)^. Therefore, further research is needed to refine and optimise these approaches for flavonoid estimation.

### Conclusion

This study concludes that the flavanols subclass was the most representative dietary flavonoid consumed by the Portuguese population. Fruit was the main contributor to the total flavonoid intake across all demographic ages. Adolescents had the lowest intakes of total dietary flavonoids. Older adult males had significantly higher intakes than adult males and less educated males had higher intakes than those with higher education levels. Females who were not physically active had significantly lower intakes than the physically active. This work provides a comprehensive understanding of flavonoid intake in the Portuguese population, identifies main food contributors and has implications for future research, policy-making and interventions to improve diets and promote health. It can also streamline forthcoming investigations into the link between flavonoid consumption and its impact on health, contributing to the future establishment of dietary reference values.

Developing a consolidated food flavonoid composition database, coded according to the FoodEx2 classification system, also represents a significant strength of our study. This facilitates the accurate estimation of flavonoids and their subclass intakes and enables the straightforward replication of our methodology across different populations. By ensuring compatibility with the FoodEx2 system, our approach offers a robust framework for adapting dietary data from various demographic groups for comparative analysis.

## Supporting information

Martins et al. supplementary material Martins et al. supplementary material

## Supplementary material

For supplementary material/s referred to in this article, please visit https://doi.org/10.1017/S0007114525000078
