# Supplementary material for: Estimated dietary flavonoid intake and major food contributors in the Portuguese population: results from the national food, nutrition and physical activity survey (IAN-AF 2015–2016)
Source: Br J Nutr. 2025 Jan 27;133(4):497–506. doi: 10.1017/S0007114525000078 (PMC12011541; doi:10.1017/S0007114525000078)
Supplement: Martins et al. supplementary material [file S0007114525000078sup001.docx]

|  | **n (%)** | **Total flavonoids** | | **Flavanols** | | **Flavonols** | | **Flavones** | | **Anthocyanidins** | | **Flavanones** | | **Isoflavones** | |
| --- | --- | --- | --- | --- | --- | --- | --- | --- | --- | --- | --- | --- | --- | --- | --- |
|  |  | **GM (95%CI)** | **p-value** | **GM (95%CI)** | **p-value** | **GM (95%CI)** | **p-value** | **GM (95%CI)** | **p-value** | **GM (95%CI)** | **p-value** | **GM (95%CI)** | **p-value** | **GM (95%CI)** | **p-value** |
| **Total** | 5005 (100.0) | 190.9 (184.0, 198.1) |  | 88.4 (82.6, 94.5) |  | 24.1 (23.3, 25.0) |  | 5 (4.7, 5.3) |  | 4.9 (4.2, 5.7) |  | 0.6 (0.4, 0.7) |  | 0.3 (0.3, 0.3) |  |
| **Sex** |  |  |  |  |  |  |  |  |  |  |  |  |  |  |  |
| Female | 2613 (51.2) | 159.9 (150.7, 169.7) | **<0.001** | 75.2 (67.5, 83.8) | **<0.001** | 22.6 (21.6, 23.6) | **<0.001** | 5.2 (4.9, 5.6) | 0.078 | 2.7 (2.2, 3.4) | **<0.001** | 0.3 (0.2, 0.5) | **<0.001** | 0.3 (0.3, 0.3) | **<0.001** |
| Male | 2392 (48.8) | 230.0 (220.4, 240.1) |  | 104.7 (98.0, 111.7) |  | 25.9 (24.6, 27.1) |  | 4.8 (4.4, 5.2) |  | 9.0 (7.3, 11.0) |  | 1.0 (0.7, 1.3) |  | 0.3 (0.2, 0.3) |  |
| **Age group** |  |  |  |  |  |  |  |  |  |  |  |  |  |  |  |
| Children | 597 (6.9) | 158.0 (146.5, 170.5) | **<0.001** | 103.0 (93.3, 113.6) | **0.041** | 17.6 (16.4, 18.9) | **<0.001** | 2.8 (2.5, 3.2) | **<0.001** | 1.8 (1.1, 2.8) | **<0.001** | 0.1 (0.1, 0.3) | **<0.001** | 0.2 (0.1, 0.2) | **<0.001** |
| Adolescences | 616 (8.4) | 158.4 (145.1, 172.8) |  | 90.4 (79.2,103.2) |  | 17.5 (16.1, 18.9) |  | 3.8 (3.4, 4.2) |  | 0.8 (0.5, 1.1) |  | 0.1 (0.1, 0.3) |  | 0.2 (0.2, 0.3) |  |
| Adults | 3091 (68.1) | 193.2 (184.7, 202.2) |  | 85.9 (79.7, 92.5) |  | 25.4 (24.3, 26.6) |  | 5.4 (5.0, 5.8) |  | 5.6 (4.7, 6.7) |  | 0.7 (0.5, 0.9) |  | 0.3 (0.3, 0.3) |  |
| Older adults | 701 (16.6) | 216.1 (192.0, 243.2) |  | 92.1 (72.6,116.7) |  | 26.1 (24.1, 28.3) |  | 5.3 (4.7,6.0) |  | 10.8 (6.8, 17.2) |  | 0.8 (0.4, 1.6) |  | 0.2 (0.2, 0.3) |  |
| **Education level** | | |  |  |  |  |  |  |  |  |  |  |  |  |  |
| None, 1^st^ and 2^nd^ cycle | 1497 (29.7) | 200.0 (186.6, 214.3) | 0.160 | 85.4 (77.7,93.9) | 0.805 | 25.9 (24.5, 27.3) | **0.002** | 4.7 (4.3, 5.2) | **0.011** | 8.4 (6.3, 11.0) | **<0.001** | 0.6 (0.4, 0.9) | 0.619 | 0.2 (0.2, 0.2) | **<0.001** |
| 3^rd^ cycle and high school | 2201 (45.8) | 182.8 (172.5, 193.7) |  | 88.3 (80.7, 96.6) |  | 22.8 (21.6, 24.1) |  | 4.8 (4.4, 5.2) |  | 3.4 (2.7, 4.2) |  | 0.6 (0.4, 0.8) |  | 0.3 (0.3, 0.3) |  |
| Higher education | 1291 (24.5) | 196.0 (178.1, 215.8) |  | 91.8 (72.9,115.7) |  | 24.6 (23.2,26.0) |  | 5.7 (5.2, 6.3) |  | 5.1 (3.6, 7.4) |  | 0.5 (0.3, 0.9) |  | 0.4 (0.3, 0.4) |  |
| **Regular physical activity (yes/no)** | | | |  |  |  |  |  |  |  |  |  |  |  |  |
| Yes | 2055 (42.4) | 203.5 (193.9, 213.5) | **0.007** | 102.5 (96.4, 109) | **<0.001** | 25.1 (23.7, 26.5) | 0.053 | 5.4 (4.9, 5.9) | **0.034** | 4.7 (3.7, 5.9) | 0.633 | 0.6 (0.5, 0.9) | 0.246 | 0.3 (0.3, 0.4) | **<0.001** |
| No | 2878 (57.6) | 182.3 (172.3, 192.9) |  | 79.4 (71, 88.9) |  | 23.4 (22.5, 24.4) |  | 4.7 (4.4, 5.1) |  | 5.1 (4.1, 6.4) |  | 0.5 (0.3, 0.7) |  | 0.2 (0.2, 0.3) |  |
| **Geographical region type** | | | | | | | | | | | | | | |  |
| PUA | 3650 (77.6) | 189.4 (181.3, 197.9) | 0.712 | 88.8 (81.9, 96.3) | 0.499 | 23.6 (22.7, 24.5) | **0.035** | 5.2 (4.9, 5.5) | 0.084 | 4.8 (4.0, 5.8) | 0.231 | 0.5 (0.4, 0.7) | 0.680 | 0.3 (0.3, 0.3) | 0.141 |
| MUA | 863 (13.8) | 197.2 (180.1, 215.9) |  | 92.0 (85.5, 99.1) |  | 27.0 (24.5, 29.8) |  | 4.4 (3.7, 5.4) |  | 4.3 (2.7, 6.7) |  | 0.7 (0.4, 1.2) |  | 0.2 (0.2, 0.3) |  |
| PRA | 492 (8.5) | 195.0 (172.0, 221.0) |  | 79.0 (60.8, 102.6) |  | 24.9 (22.3, 27.8) |  | 4.4 (3.8, 5.1) |  | 7.1 (4.6,10.9) |  | 0.7 (0.3, 1.6) |  | 0.2 (0.2, 0.3) |  |
| Abbreviations: GM – geometric mean; CI – confidence interval; PUA - Predominantly urban area; MUA - Moderately urban area; PRA - Predominantly rural area. Significant values are in **bold**. | | | | | | | | | | | | | | |  |

**Supplementary Table S1.** Mean daily dietary intake of total flavonoids and flavonoid subclasses (mg/day) by participants' characteristics, weighted for the complex survey design.
